# Metropolitan Hospital Sunday Fund

**Published:** 1906-03-10

**Authors:** 


### METROPOLITAN HOSPITAL SUNDAY FUND.

### THE INCREASE OF OUT-PATIENTS.

A meeting of the Council of the Metropolitan Hospital Sunday Fund was held on the 2nd inst. at the Mansion House, the Lord Mayor presiding.

Sir Edmund Hay Currie, the Secretary, read the report of the Committee of Distribution on the increase of out-patients. It states that on July 27 last a resolution was passed by them expressing the opinion "that the serious increase in the number of out-patients (many of whom have trivial complaints) is detrimental to the welfare of the hospitals and patients, inasmuch as they are a burden to the funds and prevent the hospital staffs from giving the necessary attention to serious cases."

A copy of this resolution was forwarded to 80 hospitals having out-patient departments, inviting the opinions of their committees on the question, and asking if they could suggest any remedy. Their replies have been received and the suggestions tabulated. It appears that the number of general out-patients is decreasing, although the attendances at the special departments cause an increase in the actual figures. Suggestions have been made to the committee that, after the first visit, patients should not be allowed to attend a second time without a certificate from a medical man in the neighbourhood that the case is a proper one for treatment at a hospital. The committee cannot see their way to agree to this proposition, but they believe that, if such cases could be referred to a private practitioner or to a self-supporting dispensary (provided sufficient of these could be established), nothing but good would result, especially if the dispensaries were attended, not by a few medical men in the district, but by the whole of the general practitioners in the immediate neighbourhood, such dispensaries to be affiliated to the nearer general hospitals.

The committee recognise that the constituents of the fund have, for the first time, decided to make grants to "district nursing associations employing fully trained hospital nurses"—there are about 300 of them engaged throughout the metropolitan area—and as these nurses work only under the direction of local medical men, the committee are of opinion that some sort of affiliation should be made, and that casualty patients might be recommended to apply to the superintendents of these associations after receiving "first aid" at the hospital, thus coming under the professional care of the local medical men.

The committee consider that, notwithstanding the large number of attendances in the out-patient departments of the general hospitals, those institutions have hitherto increased their accommodation and qualified staffs to an extent sufficient to enable them to cope with all such cases. The committee also think that there is not any serious abuse of the out-patient departments in the general hospitals, and that any which may exist at the present time can be effectually met by an efficient system of investigation, conducted by a competent staff in the out-patient department, who should visit the homes of the patients, and for that purpose the addresses of all out-patients should be taken.

The committee has considered it advisable to confine its investigation to the general hospitals only, and not to the special ones.

The report having been adopted, a resolution was passed to authorise the Council to take steps for the incorporation of the fund. It was stated that a meeting of secretaries of London hospitals, held the previous day at the Mansion House in order to revise the uniform system of accounts, had appointed a committee for the furtherance of that object.

